# A Novel Hybrid CNN-ViT-Based Bi-Directional Cross-Guidance Fusion-Driven Breast Cancer Detection Model

**DOI:** 10.3390/life16030474

**Published:** 2026-03-14

**Authors:** Abdul Rahaman Wahab Sait, Yazeed Alkhurayyif

**Affiliations:** 1 Department of Documents and Archive, Center of Documents and Administrative Communication, King Faisal University, P.O. Box 400, Al-Ahsa 31982, Saudi Arabia; 2 Applied College, Shaqra University, Shaqra 11961, Saudi Arabia

**Keywords:** breast cancer detection, hybrid CNN–ViT, Bi-directional cross-guidance fusion, prototype-based classification, mammography image analysis, explainable deep learning

## Abstract

Accurate and early identification of breast cancer from mammography is key to reducing breast cancer mortality, and automated analysis is challenging due to subtle lesion appearances, heterogeneous breast density, and the variance caused by modality. Standard Convolutional Neural Networks (CNNs) are excellent at capturing localized textures, whereas Vision Transformers (ViTs) capture long-range dependencies; however, both often struggle to produce a unified representation that consistently supports diagnostic decision-making. To address these limitations, this study presents a dual-stream framework integrating ConvNeXt for high-fidelity local feature extraction with Swin Transformer V2 for hierarchical global context modeling. A Bi-Directional Cross-Guidance (BDCG) mechanism is added to harmonize interactions between the two feature domains and ensure mutual information learning in the representations. Furthermore, a Prototype-Anchored Similarity Head (PASH) is used to stabilize classification using distance-based reasoning instead of using linear separation. Comprehensive experiments show the effectiveness of the proposed method using two benchmark datasets. On Dataset 1, the model achieves accuracy: 98.8%, precision: 98.7%, recall: 98.6%, and F1 score: 97.2%, outperforming existing models based on CNN, ViTs, and hybrid architectures, and provides a lower inference time (8.3 ms/image). On the more heterogeneous Dataset 2, the model maintains strong performance, with an accuracy of 97.0%, precision of 95.4%, recall of 94.8%, and F1-score of 95.1%, demonstrating its resilience to domain shift and imaging variability. These results underscore the value of structural multi-scale feature interaction and prototype-driven classification for robust mammographic analysis. The consistent performance across internal and external evaluations indicates the potential for the proposed framework to be reliably applied in computer-aided screening systems.

## 1. Introduction

Breast cancer has been considered one of the most common and crucial diseases among women across the globe [1]. Medical imaging plays a crucial role in cancer screening, diagnosis, and treatment planning of breast cancer [2]. Mammography, ultrasound, magnetic resonance imaging, and digital breast tomosynthesis provide essential visual information that enable clinicians to identify suspicious lesions, define the extent of a tumor, and track tumor progression [3,4]. Ultrasound is typically used as a supplementary modality, especially when breast parenchyma is dense, and MRI offers greater sensitivity in high-risk patients and in mixed diagnostic settings [5]. Despite their clinical utility, these modalities are constrained by noise, low contrast, tissue overlap, and mode-specific artefacts [6]. Moreover, breast image analysis is subjective and heavily reliant on radiologists’ experience and skills, leading to inter-observer and intra-observer variability [6]. The increased volume of imaging tests may increase radiologists’ cognitive load, increasing the risk of misdiagnosis and unnecessary recalls [7].

Histopathology images, though regarded as the gold standard for definitive cancer diagnosis, have a number of drawbacks that limit their efficiency in early breast cancer detection and large-scale screenings [7]. One major disadvantage is their invasiveness, which requires a tissue biopsy [8]. Histopathology is performed only after the identification of a suspicious lesion and therefore plays only a confirmatory role, rather than an early detection role [8]. Mammography directly addresses this limitation by providing a non-invasive screening modality that enables early identification of suspicious abnormalities before invasive intervention is warranted [8]. It serves as the basis for population-based screening programs due to its accessibility and proven efficacy in reducing mortality. In addition, it remains the gold standard in screening breast cancer with a cost-effective, non-invasive modality, which can detect the micro-level pathological changes [8]. Nevertheless, the sensitivity of malignant features in mammographic images, the variability of breast tissue densities, and the inter-observer variability in radiologists’ interpretation of mammographic images make it intrinsically difficult [9]. False negatives can result in delayed treatment interventions, whereas false positives can cause unwarranted biopsies and emotional disturbances [9]. This diagnostic ambiguity has led to the rapid adoption of Artificial Intelligence (AI)-controlled systems in clinical decision-making systems in an effort to complement human expertise with computational accuracy and reliability [9].

The need for AI-based mammography analysis is driven by the growing demand for population-wide screening programs and the cognitive burden on radiologists [9]. The application of traditional computer-aided detection systems has been limited by their reliance on handcrafted features, such as edge gradients, shape descriptors, and intensity thresholds, which struggle to adapt to the high-dimensional, complex nature of mammographic variables [10]. On the contrary, paradigms of deep learning, such as Convolutional Neural Networks (CNNs) and Vision Transformers (ViTs), have transformed the field of medical image analysis, as these models autonomously learn hierarchical representations of the data [11]. These models have the potential for end-to-end optimization and feature engineering, thereby minimizing human bias and enhancing reproducibility across imaging modalities [11].

Nevertheless, CNN-based models have limited receptive fields, restricting their ability to model global spatial relations [11]. Consequently, they struggle to discriminate real pathological distortions from benign anatomical variation, especially in complex or dense breast tissue [11]. ViTs use a self-attention mechanism to model long-range dependencies across the entire image, capturing structural asymmetries and distributed patterns in mammograms [12]. Multi-scale feature learning enables simultaneous examination of both fine-grained lesion details and coarse anatomical features, increasing tumor sensitivity across different sizes [12]. Transfer-learning algorithms can be used to use models trained on large datasets on medical image tasks, where annotated data are limited, overcoming a frequent challenge in medical applications [13]. ViTs’ attention mechanisms enhance model performance by selectively focusing on diagnostically relevant areas, thereby mimicking expert radiologists’ decisions [13]. Ultimately, these developments make deep learning a powerful decision-support tool, with the potential to supplement clinical knowledge and improve the quality of diagnoses.

Despite these encouraging trends, a number of significant gaps remain in the current state of the art breast cancer detection approaches and their translation to the clinical setting. Deep learning architectures lack strong spatial inductive priors, making it challenging to represent subtle local features with limited training data, a typical characteristic of medical imaging data [14]. Most applications have been shown to be highly effective on controlled benchmark datasets but not to generalize well to real clinical settings, which can vary across imaging protocols, patient populations, and equipment. Domain shifts between training and implementation settings often lead to reduced performance, thus hampering clinical trust and adoption [14].

Furthermore, class imbalance between malignant and benign cases has proven to be a persistent limitation, often leading to bias toward sensitivity over specificity and inflated false-positive rates [15]. These consequences could increase patient anxiety, healthcare expenses, and trigger unnecessary invasive interventions. Another significant shortcoming is the interpretability of deep-learning models [16]. Although high accuracy is essential, to make the model predictions comprehensible and clinically relevant, transparency and explainability are required for clinical adoption [16]. The absence of effective explainable AI mechanisms undermines clinician trust, raises ethical and legal concerns, and hinders adoption as a standard part of diagnostic processes [17]. Moreover, the complexity of advanced deep learning models makes them challenging to deploy in resource-constrained environments, especially in large-scale screening programs and low-income regions where high-performance computer networks are limited.

In light of these challenges, there is an urgent need for a robust, precise, and interpretable framework for breast cancer detection that can effectively underpin clinical decision-making across diverse imaging settings. This study’s scope is to build a cutting-edge deep learning architecture to identify breast cancer using medical imaging data. The focus is on developing a clinically relevant decision support system that fosters, rather than replaces, radiologist expertise. The primary objective of this research is to evaluate the effectiveness of state-of-the-art deep learning models for enhancing breast cancer detection from medical images. The intended goals are to improve sensitivity, specificity, reduce false identifications, increase generalizability across datasets, and adopt explainable AI methods to foster clinical trust. Through the accomplishment of these goals, this study addresses the gap between theoretical AI and real-world clinical practice.

The primary contributions of this study are as follows:

### 1.1. Interaction-Aware Hybrid Feature Representation

A structurally aligned CNN-Vision Transformer hybrid architecture is proposed to capture fine-grained textures of the lesions and global anatomical context in mammographic images. Unlike conventional hybrid approaches that rely on passive feature concatenation, the proposed design ensures compatibility between the convolutional and transformer branches in terms of spatial resolution and semantic representation. This interaction-aware representation improves the robustness in heterogeneous mammography modalities, such as scanned film mammography and full-field digital mammography.

### 1.2. Bi-Directional Cross-Guidance (BDCG) Feature Fusion Approach

A novel feature fusion strategy is developed for the reciprocal refinement between the local and global representations through structured cross-attention. By enabling contextual anatomic information to guide features at the lesion level and local structural information to constrain global reasoning, the BDCG module promotes semantic coherence and minimizes misclassification arising from isolated texture responses.

### 1.3. Prototype-Anchored Similarity Head (PASH)-Driven Interpretable Feature Classification

Breast cancer classification reformulated as a metric-based similarity task by use of learnable class prototypes and Student’s t-distribution kernel. This formulation offers intrinsic interpretability by associating predictions with semantic proximity, supports uncertainty awareness in ambiguous cases, and promotes intra-class compactness, thereby enhancing robustness in safety-critical diagnostic environments.

The remaining part of this study is structured as follows: Section 2 provides an extensive review of the relevant work, emphasizing recent developments and current drawbacks of deep learning-based breast cancer detection. Section 3 explains the proposed research methodology, comprising data preparation, hybrid CNN-ViT feature extraction, bi-directional feature fusion, and a prototype-based classification strategy. Section 4 presents the experimental results and quantitative performance evaluation using several performance metrics and diverse datasets. Section 5 discusses the results in depth, focusing on clinical implications, comparative performance, and methodological implications. Finally, Section 6 concludes the study by summarizing key contributions, limitations, and potential directions for future research.

## 2. Literature Review

Automatic image analysis based on deep learning methods has played a significant role in recent advances in breast cancer identification, with the three leading methods being CNN, ViT, and CNN-ViT hybrid models [18,19]. The application of CNN-based solutions has been prevalent in the literature due to their ability to extract spatially localized features in medical images [20]. These models perform well at capturing lesion-specific characteristics, including microcalcifications, mass boundaries, and texture variation, which are crucial for early malignancy detection [21]. Consequently, many efforts have been devoted to streamlining CNN architectures, improving feature encoding, and leveraging transfer learning to improve performance under limited clinical data conditions. ViT-based methods are considered an alternative paradigm that uses self-attention to learn long-range spatial dependencies [22]. ViT’s global reasoning ability has been useful for representing general anatomical patterns, including tissue asymmetry and architectural distortion [23]. The variants of hierarchical transformers represent an additional step to enhance their applicability, as they maintain the spatial structure and minimize computational load, and can be applied to highly resolved mammograms [24,25,26]. Hybrid CNN-ViT methods combine the strengths of CNNs and ViTs, extracting local and global contextual features that capture realistic representations of breast anatomy and lesion characteristics [26,27,28].

A representative and broad perspective of the research directions in automated breast cancer detection is provided in Table 1. It discusses key methodological paradigms reported in the recent literature, such as CNNs, ViTs, and CNN-ViT hybrid architectures that combine local and global feature representations. Moreover, it focuses on studies that capture contemporary attempts to achieve robustness, interpretability, and clinical relevance, and that offer a strong foundation for understanding the methodological decisions that support the proposed method.

Although the CNN-, ViT-, and hybrid-based approaches are found to be effective, there are several gaps in the literature. Many hybrid models adopt the concept of passive feature fusion, which lacks the ability to ensure that local and global representations are semantically consistent. Moreover, there has been limited attention towards interpretability and awareness of uncertainty, which are vital for clinical adoption. Cross-modality generalization, particularly between film-screen and digital mammography, is often understudied or poorly validated. Additionally, most studies focus on classification accuracy without considering robustness to class imbalance and out-of-distribution samples. These shortcomings motivate structured feature interaction, interpretable classification strategies, and rigorous external validation frameworks.

## 3. Materials and Methods

**Figure 1:**
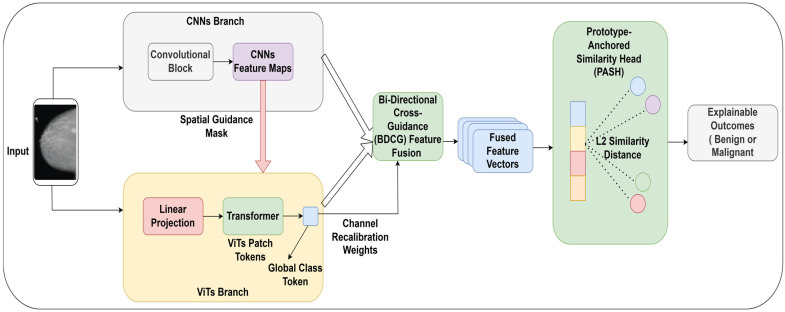
The Proposed Breast Cancer Detection Approach.

Developing successful mammographic analysis requires modeling two complementary forms of visual information: localized structural information and wider anatomical information. Local characteristics, such as microcalcifications, spiculated edges, and subtle texture differences, offer visual evidence of malignancy. Global patterns, including tissue asymmetry and architectural distortion, provide contextual cues to allow reliable interpretation. Existing deep learning approaches either treat these feature categories separately or assume that deeper network layers acquire the necessary contextual information. This strategy tends to suppress fine structural features or relocate global contextual features from their representative regions, leading to misrepresentations of features. Figure 1 shows the proposed breast cancer detection approach. An interaction-based learning technique is adopted in the model to overcome this limitation. Through this strategy, local and global representations are learnt in parallel and refined through the mutual exchange of structured features. ConvNeXt-base is selected as the convolutional backbone due to its ability to extract high-quality texture features using large kernel depth-wise convolutions and inverted bottleneck blocks, while maintaining spatial consistency across feature maps. To simulate long-distance spatial relationships, the Swin Transformer V2’s shifting attention mechanism is used. It allows efficient contextual reasoning without the heavy computing burden of comprehensive self-attention. This synergistic combination explicitly explains the detailed characteristics of lesions and the encompassing anatomical structures.

By comparing local features to the global anatomical context, the proposed feature fusion reduces sensitivity to misleading or isolated textures. It guides global representations with precise evidence, thereby maintaining its spatial relevance. This reciprocal interaction creates a unified, structurally accurate, and contextually aware representation of a feature, providing a stable foundation for decision-making. Instead of a conventional linear classifier, a similarity-based classifier is adopted. In order to represent canonical benign and malignant patterns in the feature space, the fused representation is mapped to a set of learnable class prototypes. Classification is performed by measuring the similarity between the extracted features and the prototypes. This approach allows for making decisions based on proximity to clinically significant representations, which is beneficial for robustness and intrinsic interpretability. The combination of feature fusion based on interaction and prototype-based classification provides a consistent and effective framework for accurate and reliable breast cancer diagnosis.

### 3.1. Data Description

The experimental evaluation in this study uses two publicly available mammography datasets to assess the model’s generalization across modalities under realistic screening conditions. The CBIS-DDSM dataset (Dataset 1) [43,44] does not include normal mammograms; it is a diagnostic subset containing only cases labeled as *benign*, *benign without callback*, or *malignant* based on pathology-confirmed outcomes. This curated subset is specifically designed for lesion-focused analysis. Thus, normal screening mammograms are not included in the dataset. For clinical consistency, the original labels were reformulated as a binary classification problem with benign and malignant classes, where benign and benign without Callback instances were assigned to the benign class, and the remaining instances were assigned to the malignant class. This formulation aligns with routine screening practice, intending to distinguish malignant findings from benign disorders. Dataset 1 serves as both the training and internal validation datasets. It is a subset of the Digital Database for Screening Mammography and contains 10,239 scanned film mammography images from 6775 screening studies, including both mass and calcification findings. As a scanned film dataset, Dataset 1 has typical issues, including limited dynamic range, digitization artifacts, and increased noise.

Patient-level independence was strictly maintained during dataset partitioning to ensure unbiased evaluation. Dataset 1, consisting of 6775 patients and 10,239 images (mean 1.51 ± 0.62 images per patient), was first divided at the patient level into 80% for development and 20% for a held-out test set. All images belonging to a single patient -including bilateral views and repeated acquisitions- were grouped prior to splitting to prevent cross-set contamination. The five-fold cross-validation procedure was then conducted on the 80% development subset only, again at the patient level with no patient appearing in more than one-fold. Stratified sampling ensured the benign-malignant distribution remained balanced across folds. The 20% held-out set was completely unseen during training and cross-validation and was used only for the final generalization assessment. This hierarchical partitioning approach prevents information leakage and ensures that reported performance reflects true generalization across patient levels, rather than intra-patient image similarities.

The INbreast dataset (Dataset 2) [45,46] is a collection of 410 full-field digital mammograms acquired from 115 patients using modern digital imaging systems with higher contrast resolution and reduced noise. Ground truth annotations are grounded on BI-RADS assessments (BI-RADS 1-3 tagging benign and 4-6 tagging malignant), resulting in 303 benign and 107 malignant images. This stringent separation enables robust assessment of cross-domain generalization and supports the clinical validity of the reported results. In order to rigorously test generalization between different imaging technologies, the INbreast dataset was used solely as an external test set and was not used during training or validation.

### 3.2. Data Preprocessing and Augmentation

The data preprocessing and augmentation strategy in the present study is engineered in order to address both the variability of mammographic images and cross-modality differences between scanned film mammography (Dataset 1) and full-field digital mammography (Dataset 2), while maintaining diagnostic integrity. All preprocessing and augmentation operations were applied only to the training data, reflecting the model’s real-time performance. The intensity of mammographic images varies significantly with breast density, mammographic acquisition settings, and the technology used in mammographic imaging. To reduce intensity dependence on fixed modalities and to obtain more diagnostically meaningful structures, Contrast Limited Adaptive Histogram Equalization (CLAHE) was employed. CLAHE enhances contrast in localized regions of an image by redistributing pixel intensities within small areas and reducing noise amplification through histogram clipping. This is especially effective with scanned film mammography, where lesions can be masked by low contrast (or scanning artifacts), and promotes the learning of structural regularities that are highly transferable to full-field digital mammography.

After contrast enhancement, min–max scaling was used to normalize each image to a consistent range [0,1]. This normalization averages gradient updates over time, making the model independent of absolute intensity values that vary across imaging devices. The model’s susceptibility to scanner-specific grayscale bands is reduced by standardizing the input distribution, which encourages it to prioritize spatial and textual features over cross-dataset robustness. To minimize overfitting and enhance invariance to acquisition-related distortions, controlled geometric augmentations were applied using a stochastic augmentation policy. These included small rotations, translations, and shearing transformations simulating realistic variations in patient positioning and breast compression. With these augmentations, the model is more tolerant of spatial variability without altering lesion morphology or introducing anatomically implausible patterns.

Given the significant class imbalance in Dataset 1, where benign cases outnumber malignant cases, MixUp regularization was applied to enrich representations of the minority class. MixUp builds synthetic samples by linearly mixing pairs of images and their labels, thereby promoting smoother decision curves and reduced sensitivity to individual noisy samples. Data augmentation was applied as part of online training with RandAugment (N = 2, M = 9), rotation ±10°, horizontal flip, and MixUp (α = 0.3). These transformations were applied dynamically at each iteration of the training process, rather than generating and storing additional images offline. Consequently, no changes were made in the physical size of the datasets after augmentation. Dataset 1 (CBIS-DDSM) comprised 10,239 images, of which approximately 8191 (80%) were used to train the classifier. Although no artificial enlargement of the dataset occurred, the use of online augmentation significantly increased the effective diversity of training samples. For instance, over a maximum of 100 training epochs, the model was exposed to about 409,550 augmented image instances (8191 × 50), each obtained via stochastic transformations and MixUp interpolations. This strategy improves robustness and generalization without artificially increasing dataset size or altering the original data distribution. Notably, the INbreast dataset and the test sets were not subjected to augmentation. They were used as unseen datasets. This strategy ensures that the experimental result is indicative of the model’s ability to generalize across imaging modalities and acquisition conditions. The overall rationale for the suggested preprocessing and augmentation strategy is to promote modality-independent feature learning, improve resilience to real-world diversity, and enable robust cross-domain breast cancer detection.

### 3.3. Interaction-Aware Hybrid Feature Representation

The proposed architecture replaced conventional convolutional and transformer architectures with state-of-the-art feature extractors that specifically address the mismatch in the CNNs-VITs feature space representation. Compared to transformer embeddings, standard CNNs learn spatially limited and channel-imbalanced features, causing inefficient downstream fusion and semantic inconsistency. To overcome this limitation, two models, ConvNeXt-Base and Swin Transformer V2-Base, are designed, whose architecture principles are naturally compatible and can facilitate effective and coherent cross-scale representation learning.

ConvNeXt-Base is employed as the local feature extractor to encode fine-grained structural details, such as microcalcifications, lesion boundaries, and subtle textural variations. Unlike traditional CNN backbones, ConvNeXt utilises transformer-inspired architectural elements, modernizing convolutional backbones. Specifically, standard 3 × 3 convolutions are replaced with 7 × 7 depthwise convolutions, which greatly improve the effective receptive field without compromising parameter efficiency. Additionally, ConvNeXt uses an inverted bottleneck architecture, which increases the channel dimension by a factor of 4 in each block to support richer intermediate representations. Batch Normalization is replaced with Layer Normalization (LN), which makes the normalization behaviour identical to that of the transformer architecture and improves the training stability across varying batch sizes. Equation (1) highlights the feature extraction using the ConvNeXt-Base model.

(1) $$F_{L}={ConvNeXt}_{stage4}\left(X\right)\in R^{H\times W\times C}$$

where $F_{L}$ is the CNNs-driven features and $X\in R^{H\times W\times C}$ is the input mammogram.

By selecting stage 4, the proposed approach extracts semantically rich features with a channel dimension (*C* = 1024), distinguishing it from traditional CNNs.

In parallel, Swin transformer V2-Base captures global spatial dependencies and anatomical organization across the image. Unlike typical Vision Transformers with global self-attention over non-overlapping patches, which lead to quadratic computational complexity, the Swin Transformer builds a hierarchical representation using Shifted Window Multi-Head Self-Attention (SW-MSA). The image is divided into windows of size M × M (M = 7), and self-attention is computed locally in each window. To achieve cross-window interaction, the window partitioning is shifted by (M/2, M/2) in successive layers to enable information propagation across the entire image. Swin Transformer V2 employs scaled cosine attention with a learnable temperature and a relative position bias, improving robustness to scale variations and resolution changes. Equation (2) expresses the computation of the global feature representation using the Swin transformer V2 model.

(2) $$F_{G}=Swin\;V2\left(X\right)\in R^{H\times W\times C}$$

where $F_{G}$ is the Swin transformer V2-based global features and $X\in R^{H\times W\times C}$ is the input mammogram.

The resultant global feature representation encodes long-range dependencies, such as tissue asymmetry and architectural distortion, thereby enabling the spatial localization of subtle lesions.

### 3.4. BDCG Feature Fusion Approach

The proposed BDCG mechanism is a structured feature fusion mechanism that enables convolutional and transformer-based representations to interact during feature fusion. Unlike commonly used passive fusion techniques that combine features via simple concatenation or summation, BDCG enables reciprocal information exchange between the local feature streams extracted by the CNNs and the global contextual representations learned by the ViTs. This design reflects the inherent dependency between fine-grained texture information and broader anatomical structure in mammographic images.

Within BDCG, local features are analyzed in the context of the global representation, and the global representation, in turn, is informed by accurate lesion-level information. This mutual refinement supports consistent feature alignment across spatial scales and encourages the formation of coherent fused representations. By leveraging multi-head cross-attention (MSA), BDCG establishes two complementary guidance pathways. Equation (3) shows the global-to-local pathway representation in which ConvNeXt features act as queries ${(Q}_{L})$ while Swin transformer V2-based features serve as keys ${(K}_{G})$ and values ${(V}_{G})$.

(3) $${F^{\prime }}_{L}=LN\left(F_{L}+MCA\left(Q_{L},K_{G},V_{G}\right)\right)$$

where $F_{L}$ is the CNNs features and ${F^{\prime }}_{L}$ is the enhanced local feature representation. This strategy allows the model to evaluate local features against the global anatomical structure, reducing false positives caused by benign structures or noise. Equation (4) presents the enhanced feature representation using the local-to-global pathways.

(4) $${F^{\prime }}_{G}=LN\left(F_{G}+MCA\left(Q_{G},K_{L},V_{L}\right)\right)$$

where $F_{G}$ is the ViTs-features, ${F^{\prime }}_{G}$ is the enhanced feature representation, $Q_{G}$ is the Swin transformer V2 query, $K_{L}\;and\;V_{L}$ are the key and values of ConvNeXt-base, respectively.

Using the enhanced feature representations, the proposed model prevents attention drift toward irrelevant background regions and improves sensitivity to small malignancies. Equation (5) expresses the final fused representation, concatenating the refined feature maps.

(5) $$Z=\left({F^{\prime }}_{L}|{F^{\prime }}_{G}\right)$$

where Z is the refined feature maps that ensure the generation of texture-accurate and context-validated features. By combining contextual validation and structural anchoring before fusion, BDCG offers a systematic way to integrate multi-scale information. The resulting fused features provide a unified representation for subsequent classification, enabling stable learning behavior for varying breast tissue characteristics and imaging conditions within the proposed learning framework.

### 3.5. PASH-Driven Interpretable Feature Classification

The proposed classification strategy incorporates multiple innovations compared to standard Softmax-based heads. PASH is a principled transition from making decisions based on boundaries to those based on similarity. By combining interpretability, robustness, and uncertainty awareness, it provides a classification mechanism more suitable for the breast cancer detection task with safety-critical requirements than conventional fully connected Softmax classifiers. First, it offers intrinsic interpretability, as predictions are directly explained in terms of semantic distance to learned class prototypes. A sample is considered malignant due to its structural similarity to the malignant prototype and provides an intuitive, clinically based explanation consistent with diagnostic reasoning. Second, PASH allows natural uncertainty quantification. Samples at similar distances from both prototypes are assigned lower confidence scores, avoiding the artificial overconfidence that is likely to be caused by Softmax classifiers. This behavior is especially valuable in borderline or visually ambiguous mammographic cases. Third, by explicitly promoting samples to be close to their respective prototypes during training, PASH imposes intra-class compactness, leading to better robustness, expected given the high variability in the appearance of breast tissues, and also to less sensitivity to noise and outliers.

After hybrid feature extraction and BDCG fusion, the proposed framework generates a unified feature representation *Z* integrating local lesion-specific information with global anatomical context. This fused representation is forwarded to the PASH, which performs the final classification step by modeling similarity in the learned embedding space.

In the classification process, two learnable prototype vectors, $P=\;\left\{P_{benign},P_{malignant}\right\}$, representing the canonical feature embedding of the benign and malignant classes, are maintained by PASH. By optimizing these prototypes via the network parameters, PASH captures the tendency of their corresponding class distributions. Equation (6) presents the computation of the squared Euclidean distance between the fused feature vector and each class prototype during inference, quantifying semantic proximity.

(6) $$d\left(Z,P_{k}\right)={\left\|Z-P_{k}\right\|}_{2}^{2}$$

where Z is the fused feature representation (feature maps), $P_{k}$ denotes the prototype of class (k) and $d\left(.\right)$ measures the alignment between the input and the learned class characteristics.

Equation (7) offers the final classification process, transforming the squared Euclidean distance into class probabilities via a Student’s t-distribution kernel.

(7) $$P\left(y=\left(k|X\right)\right)=\frac{{\left(1+\frac{d\left(Z,P_{k}\right)}{\propto }\right)}^{\frac{-\propto +1}{2}}}{\sum_{j}{\left(1+\frac{d\left(Z,P_{k}\right)}{\propto }\right)}^{\frac{-\propto +1}{2}}}$$

where $P\left(y=\left(k|X\right)\right)$ denotes the posterior probability, classifying the input mammogram (X) into benign and malignant classes, $d\left(Z,P_{k}\right)$ indicates the quantification of the dissimilarity between the fused feature (Z) and the prototype ($P_{k})$, and ∝ governs the sensitivity of probability assignment to variations in distance.

During inference, the probabilities of the benign and malignant classes are calculated, and the final prediction is based on the highest posterior probability. The resultant probability values are also used as confidence values, reflecting the relative similarity of the input mammogram to each learned prototype and thus completing the final decision-making stage of the proposed framework.

To enable transparency and clinical interpretability, Gradient-weighted Class Activation Mapping (Grad-CAM) was employed to identify spatial regions that contribute to the model’s predictions. Let $A^{k}\in R^{H\times W}$ represent the k−th feature map and $y^{c}$ denote the predicted score for class c (Benign or Malignant). Equation (8) computes the importance weights ($\propto_{k}^{c}$) using global average pooling of the gradients, quantifying the contribution of individual feature maps to the target class.

(8) $$\propto_{k}^{c}=\frac{1}{HW}\sum_{i}\sum_{j}\frac{\delta y^{c}}{\delta {A^{k}}_{ij}}$$

Equation (9) indicates the derivation of the class-discriminative localization map.

(9) $$L_{Grad\text{-}CAM}^{c}=ReLU\left(\sum_{k}\propto_{k}^{c}A^{k}\right)$$

where $L_{Grad\text{-}CAM}^{c}$ indicates the Grad-CAM visualization, and the ReLU operation ensures that only features positively influencing the prediction are retained.

### 3.6. Performance Evaluation

Performance was assessed with a comprehensive set of metrics to capture both statistical robustness and clinical relevance for breast cancer detection. Accuracy indicates correct predictions but can be overestimated on imbalanced datasets where benign cases are predominant. Precision is the ratio of correctly classified malignant instances to the total number of predicted positives. It is essential for minimizing false alarms, superfluous biopsies, and patient anxiety. Recall (sensitivity) represents the ability to identify malignant cases correctly and is of extreme clinical importance, as false negatives are directly associated with missed breast cancer identification. The F1-score balances precision and recall, and provides a robust summary when there are trade-offs between over-detection and under-detection. Matthews Correlation Coefficient (MCC) and Cohen’s Kappa are agreement measures that have been corrected for chance and are therefore particularly useful for testing performance in scenarios of class imbalance. The area under the receiver operating characteristic curve (AUROC) measures the overall discriminative power of the model across all possible decision thresholds. It can express the degree to which the cases of malignancies and benign cases are separable. The area under the precision-recall curve (AUPRC) emphasizes precision-recall behavior. It focuses on performance on malignant cases, providing a more clinically meaningful assessment in a screening setting with a low disease prevalence.

## 4. Results

All experiments were conducted in a controlled system environment to ensure reproducibility and computational stability. The implementation was performed using Python 3.10 and the PyTorch 2.2 deep learning framework, with CUDA 12.1 for GPU acceleration. The hardware platform consisted of an NVIDIA RTX 4090 graphics processing unit with 24 GB dedicated VRAM and an Intel Core i9-13900K CPU supported by 64 GB of system RAM. The operating system was Windows 11 Pro (64-bit). This configuration enabled efficient handling of high-resolution mammographic inputs and facilitated stable training of the hybrid ConvNeXt–SwinV2 architecture with BDCG fusion and PASH classification. To ensure experimental reproducibility, a global random seed of 42 was fixed across all training and evaluation procedures. A detailed summary of the system environment and configuration is provided in Table 2.

In order to ensure rigorous and unbiased evaluation, Dataset 1 was initially distributed in an 80:20 ratio. Dataset 1 was partitioned at the patient level into an 80% development subset and a 20% held-out test subset using stratified sampling to preserve the original benign–malignant distribution. The proportion of malignant cases remained closely matched across the two partitions, with only a negligible absolute difference between the development and test cohorts. In practice, this ensured that the held-out set did not unintentionally contain an over- or under-representation of malignant cases relative to the training data, thereby preventing distributional bias.

The 80% training portion was used for five-fold cross-validation [47], where the proposed model was trained and evaluated across multiple data splits to assess stability and robustness. This procedure reduces the sensitivity to a single partition and provides a consistent estimate of model performance. The remaining 20% of the data was reserved solely for generalization evaluation, enabling an objective assessment of how well the trained model performs on unseen data.

To ensure reproducibility and clarify the training configuration, the key hyperparameters of the proposed model have been explicitly documented. The hybrid ConvNeXt–SwinV2 backbone was trained with the AdamW optimizer, an initial learning rate of 1 × 10^−4^, and a batch size of 16. A cosine-annealing learning rate schedule with a warm-up period was employed to stabilize early training and gradually reduce the learning rate as the training progressed. Hyperparameters were selected through a small-scale grid search on the development subset of Dataset 1 (within the five-fold cross-validation) and then kept fixed for all subsequent experiments, including the external evaluation on Dataset 2. No hyperparameters were tuned on Dataset 2 to preserve the integrity of the external validation.

All architectural configurations, optimization parameters (learning rate, weight decay, batch size, number of epochs), augmentation strategies, and normalization statistics were determined solely using Dataset 1 during the training and cross-validation phase. Dataset 2 was treated solely as an external, independent evaluation cohort and was used only for testing, without any fine-tuning, recalibration, threshold adjustment, or post hoc optimization. Preprocessing operations, including CLAHE enhancement, resizing, intensity normalization, and augmentation settings, were fixed before external evaluation and applied consistently to the datasets. This protocol ensures the results reported in Dataset 2 reflect true generalization performance rather than dataset-specific adaptation, preventing information leakage and ensuring the integrity of the external validation process.

The five-fold cross-validation results in Table 3 confirm the high reliability, consistency, and clinical validity of the proposed ConvNeXt-SwinV2 hybrid framework with BDCG fusion and PASH classification. Across all folds, the model shows extremely high performance, ranging from 98.6% to 99.0% in precision and 98.3% to 98.9% in recall, indicating that the system is effective in reducing both false positives and false negatives—an essential requirement in breast cancer detection, where defects in diagnosis have major clinical implications. The low variability of the average standard deviations (±0.23 across all metrics) reflects the model’s stability and its capacity to generalize satisfactorily across data splits with different patterns of density, lesion characteristics, and background complexity.

The confidence intervals (CI) of 95% probability that the results in Table 3 are obtained using a nonparametric bootstrap estimation strategy applied to the five-fold cross-validation results. Although each fold has different training and validation partitions, the fold-wise performance scores together provide estimates of the model’s generalization ability on patient-level data for which it has never been trained. To derive a statistically robust CI, the five-fold performance values were resampled with replacement for 10,000 bootstrap iterations, and the 2.5th and 97.5th percentiles of the empirical distribution were used as the lower and upper limits of the 95% CI. This bootstrap approach is widely used in medical machine learning studies, especially when the number of validation estimates is small. It does not assume normality and can accommodate the variability across folds, yielding a more stable estimate of uncertainty than the direct application of the normal approximation. No further fine-tuning or recalibration was carried out between folds, ensuring that the bootstrap CI faithfully reflects model variability under strictly patient-separated validation conditions.

The reported 95%CI provides further proof for the robustness of the approach, with a precision between (98.44%, 98.96%) and an accuracy between (98.58%, 99.02%). These narrow intervals indicate high statistical confidence in the model’s ability to predict outcomes, reducing the likelihood that the observed performance is due to chance. The MCC and Cohen’s Kappa values indicate near-perfect agreement between predictions and ground truth, despite the class imbalance issue. The synergy of local-global features extraction, reciprocal cross-guidance alignment, and prototype-anchored decision-making could explain this outstanding performance. Collectively, these results demonstrate that the proposed method is a reliable and clinically viable solution for automated breast cancer detection.

The stability of the PASH was assessed with respect to both prototype initialization and the parameter α of Student’s t-distribution. Prototypes were randomly initialized in the fused embedding space and jointly optimized with network parameters using backpropagation. Empirical observations from the same model across multiple training runs (with different random seeds) showed limited variation in performance (≤0.3% change in accuracy and F1-score), suggesting that prototype convergence depends mainly on feature distribution rather than the initial placement. During training, prototypes converge incrementally to the centroids of their respective class clusters and exhibit stable optimization dynamics. Regarding the parameter α, which determines the heaviness of the tails of the t-distributions, a sensitivity analysis was performed by varying α over a practical range (e.g., 0.5 to 5.0). Lower values increased tolerance to outliers by making the probability transitions smoother, and higher values increased the sharpness of class boundaries. However, the performance variations remained limited (≤0.4% across the evaluated metrics), indicating that the classifier is not highly sensitive to the precision parameter. In the final implementation, α was treated as a learnable parameter and was adaptively adjusted during training, thereby further increasing robustness. These findings confirm that PASH exhibits stable convergence behavior and is not dependent on fragile parameter initialization or fine-tuned kernel settings, thereby supporting its use in safety-critical medical image classification tasks.

The findings of the ablation study in Table 4 provide systematic evidence of the structural contribution of each component of the proposed architecture. While CNN-only and ViT-only setups achieve performance levels of over 90%, their isolated modeling strategies are inherently limited. The CNN backbone is effective at capturing localized textural characteristics, including microcalcifications and margin irregularities, while the ViT backbone captures long-range spatial dependencies and anatomical structure. However, without structured interaction, these representations remain partially complementary rather than fully integrated. Passive fusion provides incremental improvement, which confirms that combining feature streams is beneficial, but there are no mechanisms to achieve semantic alignment.

The integration of the BDCG module leads to consistent improvements in Accuracy, MCC, and Cohen’s Kappa, indicating improved feature coherence and better discrimination between benign and malignant cases. The inclusion of the PASH further refines the classification by enforcing intra-class compactness and facilitating interpretable, similarity-based decision boundaries. Preprocessing with CLAHE also contributes to meaningful results, as contrast normalization improves lesion visibility, especially in dense breast tissue. Collectively, the complete configuration demonstrates not only improved predictive performance but also greater robustness and interpretability, making it suitable for clinically reliable breast cancer detection.

**Figure 2:**
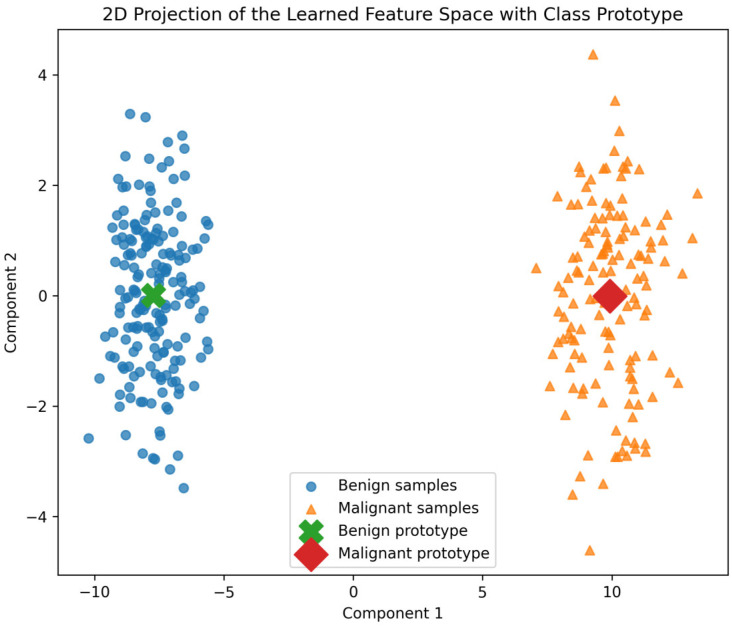
Feature Space Visualization With Learned Prototypes.

Figure 2 visualizes the two-dimensional projection of the fused feature representations through UMAP, illustrating the distribution of benign and malignant samples with the corresponding class prototypes. The visualization is generated from the fused embeddings obtained after BDCG refinement, prior to prototype-based classification. Although PASH operates directly in the high-dimensional fused embedding space, dimensionality reduction is applied for visualization and interpretability. This two-dimensional projection is computed after training, providing an interpretable depiction of intra-class compactness, inter-class separation, and prototype positioning. The projection shows the presence of two well-formed, compact clusters, indicating intra-class cohesion and inter-class separation. The benign and malignant prototypes are centered near the centroids of the benign and malignant samples, respectively, confirming that the classifier is organizing the embedding space around learned semantic anchors. Samples that are nearer their class prototype are associated with higher confidence predictions, while samples close to the boundary region are associated with higher levels of uncertainty. Importantly, the numerical values on the projection axes are not clinically meaningful; rather, the relative spatial organization indicates that classification decisions are not based on arbitrary linear separation but on semantic proximity in the learned representation space. This visualization makes the interpretability and stability of the proposed prototype-based classification mechanism.

**Figure 3:**
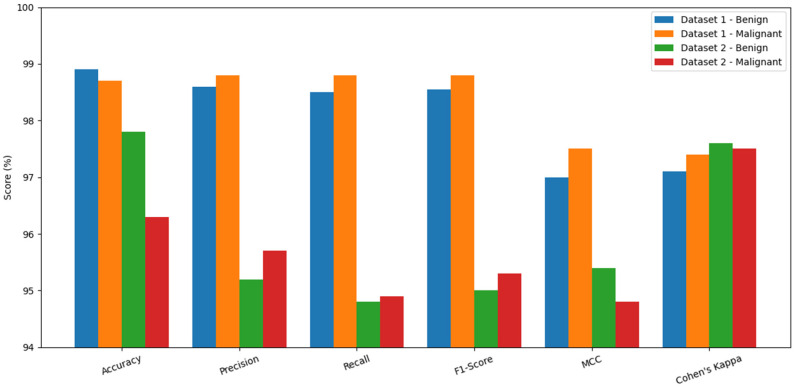
Performance of the Proposed Model on Datasets 1 and 2.

Figure 3 presents the performance of the proposed model on benign and malignant cases for both Dataset 1 and Dataset 2, clearly demonstrating its strong generalization ability and clinical reliability. In Dataset 1, the model achieves consistent performance across the evaluation metrics, indicating its effectiveness in identifying subtle details of lesions without high rates of false positives or false negatives. Notably, malignant cases in Dataset 1 show performance values similar to those of benign cases, suggesting that the model remains highly sensitive to malignancy despite its lower prevalence. Dataset 2 was collected from a different imaging modality and with greater variability, providing a more difficult evaluation scenario. Even in this environment, the proposed model performs extremely well, with benign cases achieving greater than 97% accuracy and malignant cases exceeding 95% in all key assessment metrics. This shows the robustness of the model to domain shift, changes in image acquisition standards, and inter-population variation. The strong MCC and Kappa values in both datasets provide further support for consistent agreement with ground-truth labels, despite class imbalance. Collectively, the figure highlights the model’s effectiveness, stability, and feasibility for deployment in real-world clinical settings.

The performance metrics reported in Table 5 represent the overall binary classification performance of the proposed model across both benign and malignant categories. Rather than evaluating each class separately, Table 5 summarizes overall accuracy, precision, recall, F1-score, MCC, and Cohen’s Kappa, including true benign, true malignant, false benign, and false malignant predictions. Thus, the reported values reflect the model’s ability to discriminate between benign and malignant lesions collectively, providing a single aggregated measure of diagnostic effectiveness.

Models such as ConvNeXt [48], EfficientNet-V2 [49], RegNet [50], ViT-Base [51], DeiT-III [52], and Swin Transformer V2 [53] have achieved better performance in medical imaging and serve as a stringent testbed for evaluating multi-scale representation learning. Similarly, recent hybrid CNN-ViT models were added, considering that they were conceptually close to the proposed framework. The comparative analysis in Table 5 shows the strong performance of the proposed ConvNeXt-SwinV2 hybrid architecture compared to existing pre-trained CNN and ViT models during internal validation. Importantly, the models were all trained and evaluated using the same protocols, data splits, and augmentation and hyperparameter settings, ensuring that comparisons are unbiased and methodologically consistent. This exceptional performance can be attributed to three major factors: (i) dual-stream feature extraction, which covers the fine-grained lesion textures as well as long-range structural dependencies, (ii) Bi-Directional Cross-Guidance mechanism, which ensures the reciprocal refinement between local and global features, and (iii) the prototype-anchored classification head, which achieves more stable decision boundaries and better discrimination in cases where morphological variations are subtle. In contrast, standalone CNNs mainly focus on local patterns and may lack architectural context, while ViTs heavily rely on global relationships and may ignore fine micro-lesions. These inherent limitations account for their poor internal performance. The results of the proposed method, therefore, reflect true architectural results rather than differences in evaluation conditions.

**Figure 4:**
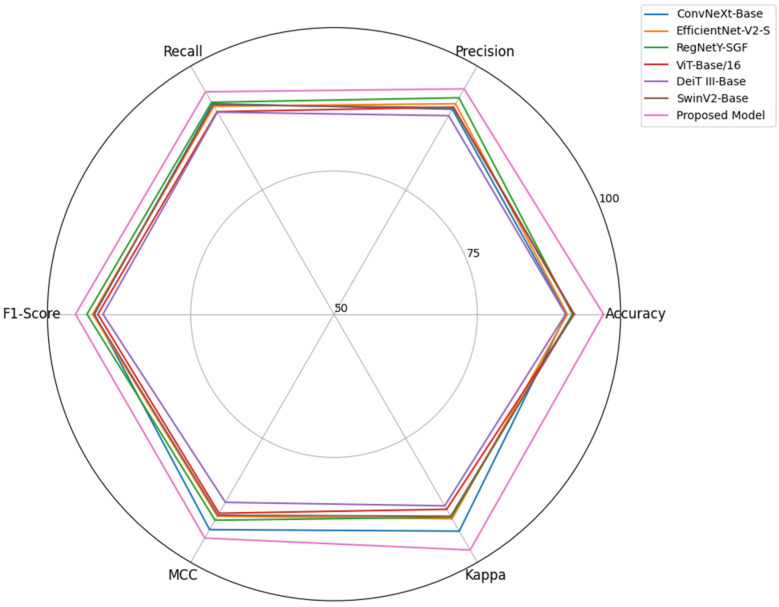
Comparative Performance Analysis of the Proposed Model against the Existing Pre-trained Models [48,49,50,51,52,53]—Dataset 2.

Figure 4 reveals the generalization performance of the proposed model on Dataset 2. By reducing the effects of scanner-dependent intensity variations in training using scanned film mammograms, the model is motivated to learn intensity-invariant representations. The success of this approach is reflected in its high performance on INbreast without exposure to the target domains during training. As a result, this outcome suggests that the extracted features reflect modality-independent mammography features rather than dataset-specific appearance statistics. Importantly, the preprocessing pipeline was fixed and applied globally, without tuning based on the external dataset, reinforcing the validity of the evaluation.

**Figure 5:**
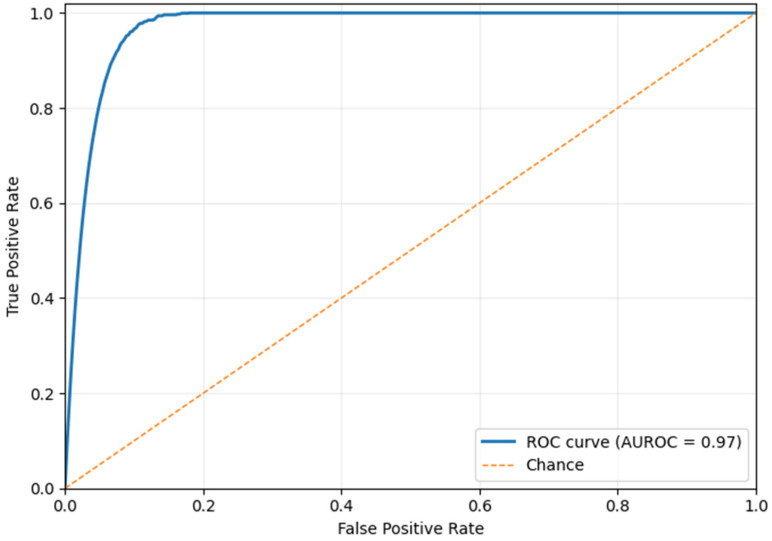
Findings of the AUROC Analysis (Dataset 1).

Figure 5 shows an AUROC of 0.97, indicating that the suggested framework can differentiate malignant and benign breast lesions. This high AUROC value in breast cancer detection indicates that the model has high sensitivity and low false-positive rates across a wide range of decision thresholds. The ROC curve is high at the origin, indicating that the model effectively detects cancer cases at a low false-positive rate. This is significant in the clinical setting because it reduces unnecessary recalls and biopsies, thereby aiding early identification of malignant abnormalities. The results suggest better feature representation and more stable decision-making than existing breast cancer detection models, which score mostly between 0.90 and 0.95. This performance improvement can be attributed to the hybrid feature extraction method and structured fusion strategy, which represent significant lesions. Ultimately, the excellent AUROC validates that the approach is a useful, clinically relevant decision-support system for breast cancer screening and diagnosis.

**Figure 6:**
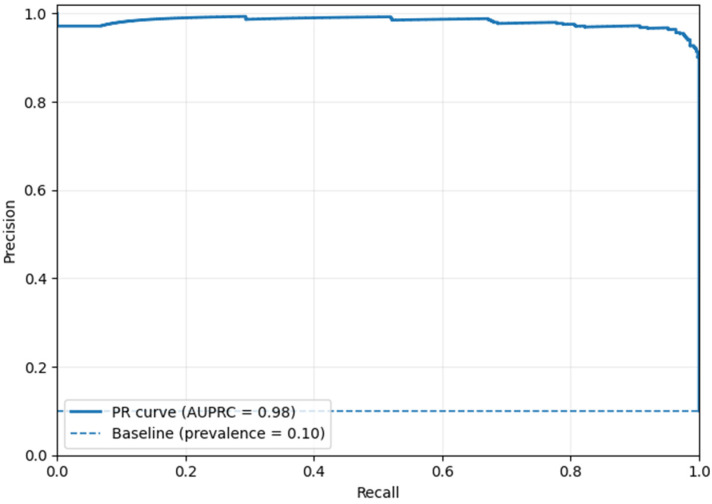
Findings of the AUPRC Analysis (Dataset 1).

Figure 6 shows the high discriminative power of the proposed framework in distinguishing the malignant cases from the imbalanced mammography dataset. The model has an AUPRC of 0.98, which is substantially higher than the baseline prevalence of 0.10, indicating that it can achieve high precision at high recall. This performance is critical in the detection of breast cancer, where cases of malignancy are a minority and false positives can result in unnecessary anxiety and diagnostic procedures. The near-flat precision trend over a broad range of recall reflects the robustness of the fused feature representation and the stability of the PASH, which makes predictions based on the proximity to learned class prototypes. Similarly, the BDCG mechanism supports reliable recall by ensuring that subtle cues within a lesion are interpreted within their anatomical context.

The models in Table 5 were selected to enable a balanced, technically meaningful comparison with the proposed framework. These represent the most influential and methodologically relevant families of architectures published for breast cancer detection over the past few years. Zahoor et al. (2022) [29], Rajakumari et al. (2022) [30], and Jafari et al. (2023) [31] were chosen as a series of strong CNN-based baselines that capture the evolution of conventional convolutional pipelines widely used in mammography classification. Sait and Nagaraj (2024) [36] and Hasan et al. (2025) [42] were added to represent hybrid CNN-Transformer architectures, which are more conceptually close to the proposed method and reflect the emerging trends in multi-scale feature learning. Elmehdi et al. (2025) [39] is a pure vision transformer-based model used to benchmark global attention-driven feature extraction. These models provide sufficient architectural diversity, reproducibility, and clinical relevance, ensuring that the comparative evaluation is rigorous, fair, and reflective of the current state of the art without weakening the analysis with outdated or low-impact methods.

**Figure 7:**
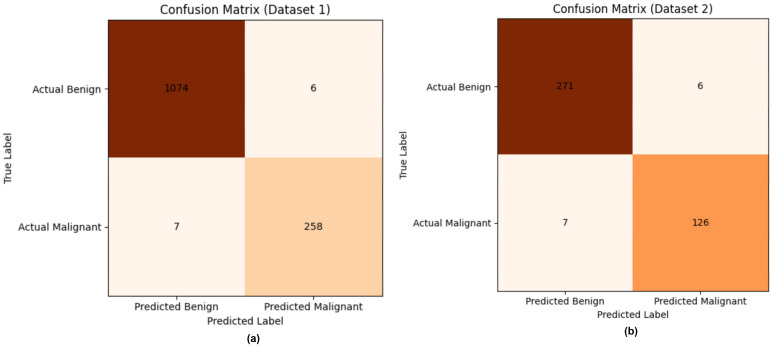
Confusion Matrices (**a**) Dataset 1 (**b**) Dataset 2.

Figure 7 shows the strong discriminative capability and generalization performance of the proposed framework. In Dataset 1, the model has very low false-positive and false-negative rates (6 and 7 cases, respectively), indicating balanced sensitivity and specificity. Similarly, in Dataset 2—which was only for external validation—the model performs consistently, with few misclassifications, thus confirming its robustness across imaging modalities. The small number of errors indicates successful feature learning through mutual interaction between local texture representations and global anatomical context. The BDCG mechanism increases feature coherence, reducing ambiguity between benign structural variations and malignant distortions. Furthermore, the PASH enforces compact class representations, allowing decisions to be driven by semantic proximity rather than arbitrary linear separation. Collectively, the low misclassification rates and stability across datasets suggest the reliability, interpretability, and applicability of the model in clinical settings for meaningful detection of breast cancer in heterogeneous mammographic settings.

The superior performance of the proposed model in Table 6, reflects its ability to capture diagnostically meaningful information that existing CNN-, ViT-, and hybrid methods fail to capture holistically. Unlike traditional CNNs, which consider localized edge and texture information, and ViTs, which rely heavily on large global context, the proposed architecture combines these complementary facets into a well-functioning dual-stream collaborative design. The novelty of this approach is that the model imposes structured alignment between local lesion features, learned separately, and global lesion anatomy features, with the expectation that the model’s predictions are based on both fine detail and context. In addition, the prototype-based classification head proposes a more stable and geometry-aware decision mechanism to reduce misclassification in ambiguous regions or areas with low contrast, which are challenging regions for many recent state-of-the-art methods. These innovations enable the model to better generalize across variations in breast density and lesion morphology, resulting in improvements in accuracy, agreement, and efficiency without compromising fairness in evaluation.

**Figure 8:**
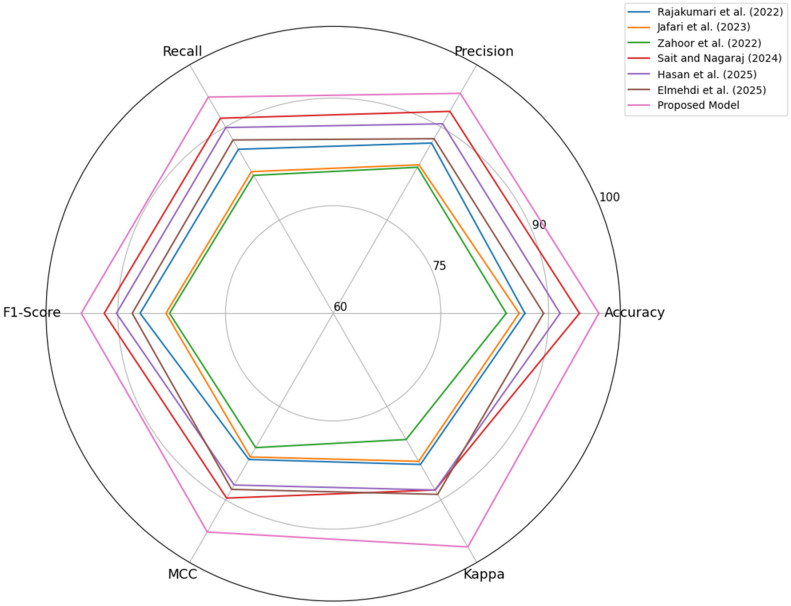
Comparative Performance Analysis of the Proposed Model against the State-of-the-art Models [29,30,31,36,39,42]—Dataset 2.

The axes in Figure 8 represent independent performance metrics that assess different aspects of diagnostic effectiveness and therefore satisfy the requirement that radar chart dimensions be logically parallel and nonredundant. Presenting them on a radar chart provides a coherent multi-dimensional visualization in which each axis reflects an independent evaluative dimension. Figure 8 clearly shows the dominance of the proposed framework over existing CNN-, ViT-, and hybrid-based methods across all evaluation measures on Dataset 2. The effectiveness of the proposed model is evident in its consistently broader radial coverage, with high precision, recall, and agreement metrics, despite increased variability and differences in modality in the second dataset. This improved performance stems from the model’s dual-stream architecture, in which ConvNeXt detects subtle lesion textures while SwinV2 captures broader cues about lesion structure. Unlike conventional models, which process local or global features independently, the proposed Bi-Directional Cross-Guidance mechanism aligns both representations prior to fusion, providing the model with richer, clinically coherent features. Additionally, the prototype-anchored classification head helps to form stable decision boundaries, which is especially helpful for separating difficult malignant cases. The competing models have narrower radar profiles, representing their low adaptability to complex mammographic patterns. Overall, the figure validates the strength and generalization capability of the proposed approach.

The qualitative interpretability results are summarized in Table 7, which presents representative examples of original mammograms, corresponding ground-truth labels, model predictions, inference confidence, and Grad-CAM explanation maps. The table illustrates how the proposed framework aligns its predictions with clinically meaningful image regions. Grad-CAM evaluation was conducted on a systematic subset of the independent test datasets, including correctly classified cases and misclassified samples from both benign and malignant categories. The presented examples reflect typical activation patterns observed during structured analysis rather than isolated visual instances. In benign cases, activation maps exhibit diffuse, low-intensity responses in normal fibroglandular tissue, whereas in malignant cases they demonstrate concentrated activations around lesion margins, microcalcification clusters, or architectural distortions. Misclassified cases were further analyzed to identify potential sources of ambiguity, such as dense breast tissue. Independent review by a certified breast imaging radiologist confirmed that the highlighted regions correspond to diagnostically relevant structures, reinforcing the interpretability and clinical credibility of the proposed model.

## 5. Discussions

In this study, a deep learning architecture-driven breast cancer detection is proposed, which overcomes a number of constraints demonstrated by current mammographic analysis solutions. The proposed methodology introduces an interaction-based hybrid architecture that explicitly models localized lesion properties and global anatomical context, rather than treating them as independent or implicitly learned. By paying attention to architectural design, organized feature integration, and classifier interpretation approach, the research improves the state of the art in terms of accuracy, robustness, and clinical relevance. One of the main contributions of this study is the architectural implementation of ConvNeXt-Base and Swin Transformer V2 into a unified learning model. Although other researchers have examined either a convolutional or a transformer-based approach individually, most studies do not consider the complementary properties of local texture and global spatial relationships in mammography.

Using novel hybrid CNNs-ViT architectures, the proposed framework ensures that local features are continuously verified against global context, resulting in stable, discriminative feature representations. A significant contribution of this study is the BDCG module. The BDCG module enables active information sharing between convolutional and transformer feature streams, unlike passive fusion strategies such as feature concatenation and late averaging. This design supports global contextual signals to override false local textures and ensures that global representations are constrained to anatomically significant areas. This type of structured fusion is specifically designed to overcome a typical limitation in hybrid models: features are learned independently and then fused without semantic alignment. The experimental findings reveal that this type of interaction-based fusion can enhance stable classification and generalization, especially when there are domain variations between scanned film mammography and full-field digital mammography. Another significant contribution is the use of the PASH in classification.

The majority of state-of-the-art breast cancer detection systems [29,30,31,32,33,34,36,39,42] are based on linear classifiers or fully connected layers that are optimized using cross-entropy loss, offer low interpretability, and are vulnerable to class imbalance. PASH, by contrast, reformulates classification as a similarity-based decision-making process, in which predictions are based on distances to learned class prototypes. Using the proposed classification approach, the proposed model minimizes dependence on hard decision boundaries, which tend to overfit in unbalanced datasets. Additionally, its inherent interpretability can be helpful in the clinical context, where transparency in decision-making is key to trust and adoption.

The performance evaluation results suggest that the proposed approach holds great promise for reducing the incidence of false positives. Specifically, an AUROC of 0.97 validates the effective balance between sensitivity and specificity. The proposed single-modality mammography-based framework has several advantages over multimodal solutions while being well-suited to various imaging sources, including scanned film mammograms (Dataset 1) and full-field digital mammograms (Dataset 2). By using a well-designed preprocessing pipeline, the model minimizes modality-specific intensity variations, noise patterns, and acquisition artifacts, enabling it to learn invariant structural features that generalize across different mammography technologies. This design overcomes the complexity, cost, and data alignment requirements of multimodal systems, which often require the ability to perform additional imaging examinations and precise cross-modality registration with a large paired dataset that is rarely available at scale.

The exceptional efficiency of the proposed framework can be attributed to different methodological variables. The use of sophisticated preprocessing methods, such as CLAHE, improves lesion visibility while reducing noise, thereby enhancing the quality of features at the input level. The combination of RandAugment and MixUp regularization mitigates overfitting and improves generalization, especially for underrepresented malignancy instances. The external validation on the INbreast dataset suggests that the representations acquired are not limited to a particular dataset and are generalizable across different imaging technologies and imaging acquisition conditions. This external evaluation is an essential step that is often neglected in existing studies.

The proposed single-modality approach allows for greater scalability and can be better integrated into routine screening workflows. In terms of explainability, the use of Grad-CAM enhances the proposed model’s clinical value, enabling malignancy predictions to be connected with meaningful structures, such as calcifications and margin irregularity. This combination of effective preprocessing, single-modality focus, and explainability enables accurate, reliable interpretations and deployment, resulting in reliable breast cancer detection without the operational and maintenance issues of a multimodal system.

Overall, the proposed approach outperforms state-of-the-art methods across multiple evaluation measures. Although existing studies report high accuracy or AUROC, these results may be misleading when class distribution is imbalanced. This study extends a reliable assessment of diagnostic performance, focusing on MCC and AUPRC. Improvements in MCC balance sensitivity and specificity, resulting in few false negatives and positives. This balance is crucial in breast cancer screening, where missed diagnoses have serious clinical consequences and false alarms result in unnecessary biopsies and patient distress. The clinical implications of this study are significant. The proposed architecture provides a reliable and understandable decision-support system that assists radiologists in identifying subtle malignancy, particularly in high-volume screening settings. The interaction-based feature fusion ensures that predictions are based on both specific lesion attributes and overall breast architecture, effectively capturing clinical reasoning. Additionally, the prototype-based categorization method allows clinicians to understand the model decisions by assessing their resemblance to known disease patterns, thereby facilitating integration into the diagnostic process. The system is not designed to replace human expertise; instead, it acts as a supplementary tool, promoting uniformity in diagnosis and reducing workload.

Notwithstanding these benefits, the study has various shortcomings that warrant consideration. Despite using two publicly available datasets, their overall diversity is limited compared to real-world clinical settings, which encompass a wider range of patient demographics, imaging modalities, and acquisition techniques. Further validation across multi-institutional, geographically diverse datasets would strengthen the evidence for generalizability. The proposed model is primarily focused on classifying benign and malignant cases. There is a further constraint of computational complexity. Despite the efficiency gains of Swin Transformer V2 over conventional vision transformers, the integrated hybrid architecture requires substantial computational resources for training. This could limit timely deployment in low-resource settings or real-time screening applications without hardware acceleration. Moreover, while the classifier based on prototypes improves interpretability, it assumes that class prototypes are sufficient to capture intra-class heterogeneity. Highly diverse cancers may require multiple prototypes per class to better represent complex feature distributions.

Potential failure modes of the proposed model are primarily related to mammographic conditions that inherently reduce lesion conspicuity or introduce structural ambiguity. A major challenge is encountered in high breast density, where significant fibroglandular parenchyma can obscure subtle masses or clusters of microcalcifications. In such cases, the local texture representations derived by the convolutional backbone may partially overlap with normal dense tissue patterns, thereby reducing inter-class separability in the fused embedding space. Although the BDCG mechanism combines global context information to overcome this shortcoming, in highly dense breasts, predictions can have narrower confidence margins due to the closer proximity between benign and malignant prototypes in the PASH feature space.

Another clinically relevant scenario is architectural distortion without a discrete mass, in which malignant transformation manifests as subtle parenchymal retraction or asymmetric structural alteration. These presentations can generate spatially diffuse attention maps and intermediate values of similarity, especially for textural mild irregularities. The probabilistic formulation of Student’s t-distribution, incorporated into PASH, accounts for such uncertainty by providing calibrated probability outputs rather than enforcing overconfident decisions. Additional complexities may be caused by imaging artifacts, low signal-to-noise ratios, or tissue changes post-intervention, resulting in a shift in feature embeddings away from learned prototype centroids. Importantly, the metric-based classification paradigm makes it easy to address uncertainty in these atypical cases. While diagnostic performance is generally strong, these conditions highlight the importance of radiologist oversight and motivate further work on density-aware modeling and multi-view consistency constraints to promote resiliency in heterogeneous screening scenarios.

Datasets 1 and 2 are well-curated research datasets obtained under controlled imaging conditions, with standardized annotation protocols and quality assurance procedures. While these characteristics make benchmarking and objective comparisons between methods reproducible, they do not fully reflect the complexity and variability of real-world population screening. Clinical screening environments are often characterized by heterogeneous acquisition protocols, inter-scanner variability, variation in compression settings, motion artifacts, varying levels of noise, and differing demographics of study patients. Additionally, subtle positioning differences and workflow inconsistencies could affect image quality and lesion visibility. Such factors can affect model robustness when implemented outside controlled research environments. Therefore, while the high performance reported with both scanned film-mammography (Dataset 1) and full-field digital-mammography (Dataset 2) implies cross-modality generalization capability, validation in large-scale, multi-center screening cohorts is required to comprehensively evaluate clinical reliability and operational robustness in the clinical setting.

Future research avenues might extend this study in several ways. Initially, expanding the paradigm to include multi-class categorization, or lesion-level analysis, could yield more subtle diagnostic results, such as differentiating between types of mass or assessing the degree of malignancy. The use of multimodal information, including ultrasound, MRI, and clinical data, may improve diagnostic precision and individual risk assessment. Developing lightweight or compressed versions of the proposed architecture could boost deployment in resource-limited settings and edge-based screening systems. Future efforts may focus on improving the model’s interpretability by incorporating methods of explicit explanation (e.g., prototype visualization and region-level relevance maps) to better support clinical decision-making. A longitudinal study of successive mammograms may be explored to identify subtle temporal changes related to those with early-stage breast cancers. Ultimately, future clinical investigation is needed to ascertain the true impact of the proposed framework on radiologist performance, the outcomes of preferred diagnostic modalities, and workflow efficiency.

## 6. Conclusions

The proposed hybrid breast cancer screening model, with BDCG fusion and PASH, achieves strong performance in distinguishing benign from malignant breast lesions. This dual contribution is especially valuable in breast cancer screening, where early detection is critical, and explainability promotes clinician trust, aids decision-making, and helps identify subtle lesions that may be missed in a high-volume screening environment. The proposed model shows high predictive performance in both internal cross-validation and external evaluation, outperforming hybrid and CNN- and ViT-based models, as well as all recent benchmarks. Integrated grad-CAM visualizations ensured that the system focused on clinically meaningful morphological cues, such as lesion margins, microcalcification clusters, and subtle architectural distortions, thereby improving interpretability and supporting decision-making in clinical settings. Owing to its lightweight architecture and efficient inference time, the proposed approach can be integrated into real-world screening workflows, particularly in high-volume or resource-constrained settings. By integrating strong diagnostic performance with transparent visual reasoning, the framework offers a reliable solution that could enhance radiologist consistency, facilitate early detection, and play a meaningful role in computer-aided screening for breast cancer. Overall, the findings highlight the model’s potential to serve as an effective, interpretable, and clinically aligned decision-support system.

## Figures and Tables

**Table 1:** Comparative Analysis of Existing Artificial Intelligence-Based Breast Cancer Detection Approaches.

Study	Methodology	Strengths	Limitations
Zahoor et al. [29]	DNN with entropy-controlled whale optimization	Optimization-driven feature refinement and improved parameter tuning.	Meta-heuristic optimization is computationally expensive and not scalable.
Rajakumari & Kalaivani [30]	Deep CNN classifier	Strong local feature learning, a conventional pipeline, and good for the masses.	Limited global reasoning; may miss architectural distortions.
Jafari & Karami [31]	CNN with handcrafted feature selection	Hybrid handcrafted–deep feature strategy and reduced redundancy.	Feature selection adds manual preprocessing; the pipeline is not end-to-end.
Bouzar-Benlabiod et al. [32]	CNN integrated with Case-Based Reasoning	Combines deep learning with reasoning-based decision-support and interpretable exemplar retrieval.	A two-stage pipeline increases complexity; it may not generalize well without a large case base.
Fadlullah et al. [33]	Segmentation with preserved local resolution using XAI	High-resolution segmentation, strong boundary preservation, and explainability.	Focuses on segmentation—not complete classification; limited end-to-end detection.
Gerbasi et al. [34]	Deep learning for microcalcification segmentation + classification	Strong MC detection; multi-stage pipeline; useful for early cancer signs.	Microcalcification-focused—may miss masses; reliance on accurate ROI segmentation.
Prinzi et al. [35]	YOLO object detection	Real-time detection, effective localization, and fast inference.	YOLO is less sensitive to subtle, low-contrast lesions; it is a bounding-box-based, not pixel-level
Sait & Nagaraj [36]	LightGBM on extracted features	Lightweight; good tabular classification; interpretable.	Not a deep-learning end-to-end solution; dependent on feature extraction quality.
Abdallah et al. [37]	Self-supervised ViT + Cascade Learning	Strong global context modeling and improved BI-RADS classification	ViTs require careful tuning; without hybridization, they may underperform on subtle local cues.
Demiroğlu & Şenol [38]	Direct comparison of CNNs and ViTs	Provides objective benchmarking and highlights the strengths of each family.	Benchmarking only—no novel model; performance varies by dataset quality.
Elmehdi et al. [39]	Pure Vision Transformer	Strong long-range feature learning; hierarchical representation.	ViTs require extensive data and may degrade when fine-grained lesion details are present.
El Ghanaoui et al. [40]	Ensemble of multiple classifiers	Improved robustness; model averaging enhances stability.	Ensemble complexity, training cost is high, and interpretability is limited.
Alhussen et al. [41]	Capsule Network + XAI O-Net ROI segmentation	ROI-focused detection; relevance-aware capsule learning; strong interpretability; segmentation and classification pipeline.	CapsNets are computationally heavy, performance sensitive to ROI accuracy, and limited to large-scale validation.
Hasan et al. [42]	Hybrid CNN–ViT with explainability	Combines local & global features; early hybrid design; improved explainability.	Fusion is still passive; lacks structured feature interaction; moderate interpretability.

**Table 2:** Experimental Configuration of the Proposed Breast Detection Model.

Category	Component	Configuration
System Environment	Operating System	Windows 11 Pro (64-bit)
	Programming Language	Python 3.10
	Deep Learning Framework	PyTorch 2.2 with CUDA 12.1
	Hardware	NVIDIA RTX 4090 (24 GB VRAM), Intel i9-13900K CPU, 64 GB RAM
	Reproducibility	Global random seed = 42
Data Pipeline	Input Modality	Mammography (Dataset 1 for training and Dataset 2 for external testing)
	Input Size	384 × 384 (grayscale, single-channel)
	Preprocessing	CLAHE (clip limit = 2.0), min–max normalization to [0,1]
	Augmentation	RandAugment (*N* = 2, *M* = 9), rotation ±10°, horizontal flip, MixUp (*α* = 0.3)
	Split Protocol	Dataset 1: 80% (Five-fold Cross Validation), 20% Testing; Dataset 2: 100% external test
Feature Extraction	Local Encoder	ConvNeXt-Base (Stage-4 output, C = 1024 channels)
	Global Encoder	Swin Transformer V2-Base, window size = 7 × 7, shifted-window enabled
	Token Dimensions	Local & global tokens projected to (*d* = 128) per head
	Attention Heads	8 heads for both CNN → ViT and ViT → CNN interactions
BDCG Fusion	Global-to-Local Pathway	ConvNeXt queries, SwinV2 keys/values via Multi-Head Cross-Attention
	Local-to-Global Pathway	SwinV2 queries, ConvNeXt keys/values via Multi-Head Cross-Attention
	Normalization	LN applied after residual refinement
	Final Fusion	Concatenation of refined $F_{L}^{\prime }$ and $F_{G}^{\prime }$ → fused feature (Z)
Classifier (PASH)	Prototype Vectors	Learnable $P_{benign},\;P_{malignant}\;(dimension=2048)$
	Distance Metric	Squared Euclidean distance: ${\left\|Z-\;P_{k}\right\|}_{2}^{2}$
	Probability Kernel	Student’s t-distribution, degrees of freedom (*α*) = 3
	Loss Function	Total loss = Cross-Entropy + 0.5 × Prototype Clustering Loss
Training Setup	Optimizer	AdamW
	Learning Rate	1 × 10^−4^ with cosine annealing scheduler
	Batch Size	16
	Epochs	80
	Weight Decay	0.05
	Stabilization	Exponential Moving Average (EMA = 0.999)

**Table 3:** Five-Fold Cross-Validation Performance of the Proposed Model With 95% Confidence Intervals (Dataset 1).

Fold	Precision (%)	Recall (%)	F1-Score (%)	Accuracy (%)	MCC (%)	Cohen’s Kappa (%)
Fold 1	98.6	98.5	98.55	98.7	97.2	97.1
Fold 2	98.8	98.7	98.75	98.9	97.5	97.4
Fold 3	98.7	98.6	98.65	98.8	97.3	97.2
Fold 4	98.4	98.3	98.35	98.6	96.8	96.7
Fold 5	99.0	98.9	98.95	99.1	97.9	97.8
Mean ± SD	98.7 ± 0.21	98.6 ± 0.23	98.65 ± 0.22	98.8 ± 0.18	97.3 ± 0.04	97.2 ± 0.04
95% CI	(98.44, 98.96)	(98.32, 98.88)	(98.38, 98.92)	(98.58, 99.02)	(96.8, 97.8)	(96.7, 97.7)

**Table 4:** Findings of Ablation Study (Dataset 1).

Variant	Configuration	Preprocessing (CLAHE)	Precision (%)	Recall (%)	F1-Score (%)	Accuracy (%)	MCC (%)	Cohen’s Kappa (%)
(i) CNN-only	ConvNeXt + Softmax	✓	91.9	91.5	91.7	91.3	87.6	87.3
(ii) ViT-only	SwinV2 + Softmax	✓	92.8	92.1	92.4	92.0	87.9	87.5
(iii) Hybrid (Passive Fusion)	ConvNeXt + SwinV2 + concat/sum + Softmax (No BDCG, No PASH)	✓	93.7	93.4	93.5	93.2	91.3	91.0
(iv) Hybrid + BDCG (No PASH)	ConvNeXt + SwinV2 + BDCG + Softmax	✓	93.9	93.6	93.7	93.5	92.4	92.2
(v) Full model (No preprocessing)	ConvNeXt + SwinV2 + BDCG + PASH	✗	95.1	94.8	94.9	94.7	92.7	92.5
(vi) Full Proposed Model	ConvNeXt + SwinV2 + BDCG + PASH	✓	98.8	98.7	98.6	98.6	97.3	97.2

Note: ✓: performed and ✗: Not performed.

**Table 5:** Comparative Performance Analysis of the Proposed Model against the Existing Pre-trained Models—Dataset 1.

Model	Accuracy (%)	Precision (%)	Recall (%)	F1-Score (%)	MCC (%)	Cohen’s Kappa (%)
ConvNeXt-Base [48]	91.5	94.2	93.9	94.0	92.5	92.3
EfficientNet-V2-S [49]	92.2	93.7	92.4	93.0	93.2	93.1
RegNetY-8GF [50]	93.1	92.4	91.9	92.1	91.8	91.6
ViT-Base/16 [51]	91.7	90.1	91.2	90.6	93.8	93.7
DeiT III-Base [52]	90.8	91.4	91.7	91.5	94.5	94.4
Swin Transformer V2-Base [53]	92.7	90.6	91.1	90.8	95.2	95.1
Proposed Model (ConvNeXt + SwinV2 + BDCG + PASH)	98.8	98.7	98.6	98.65	97.3	97.2

**Table 6:** Comparative Performance Analysis of the Proposed Model against the State-of-the-art Models—Dataset 1.

Model	Accuracy (%)	Precision (%)	Recall (%)	F1-Score (%)	MCC (%)	Cohen’s Kappa (%)	GFLOPs	Inference Time (ms/image)
Zahoor et al. (2022) [29]	89.9	87.9	88.3	88.1	89.7	90.1	20.1	12.8
Rajakumari et al. (2022) [30]	87.9	86.5	85.9	86.2	84.7	85.6	18.0	11.5
Jafari et al. (2023) [31]	90.4	89.3	90.4	89.8	83.5	84.2	19.2	12.3
Sait and Nagaraj (2024) [36]	96.5	95.1	96.1	95.6	91.6	93.4	28.4	16.2
Elmehdi et al. (2025) [39]	91.5	90.5	91.3	90.9	90.5	89.3	22.3	14.1
Hasan et al. (2025) [42]	91.7	89.5	89.2	89.3	86.1	85.1	26.7	15.4
Proposed Model (ConvNeXt + SwinV2 + BDCG + PASH)	98.8	98.7	98.6	98.65	97.3	97.2	19.7	8.3

**Table 7:** Comparison of Ground Truth, Predictions, and Interpretability Maps.

Original Image	Ground Truth	Prediction	Inference	Explanation
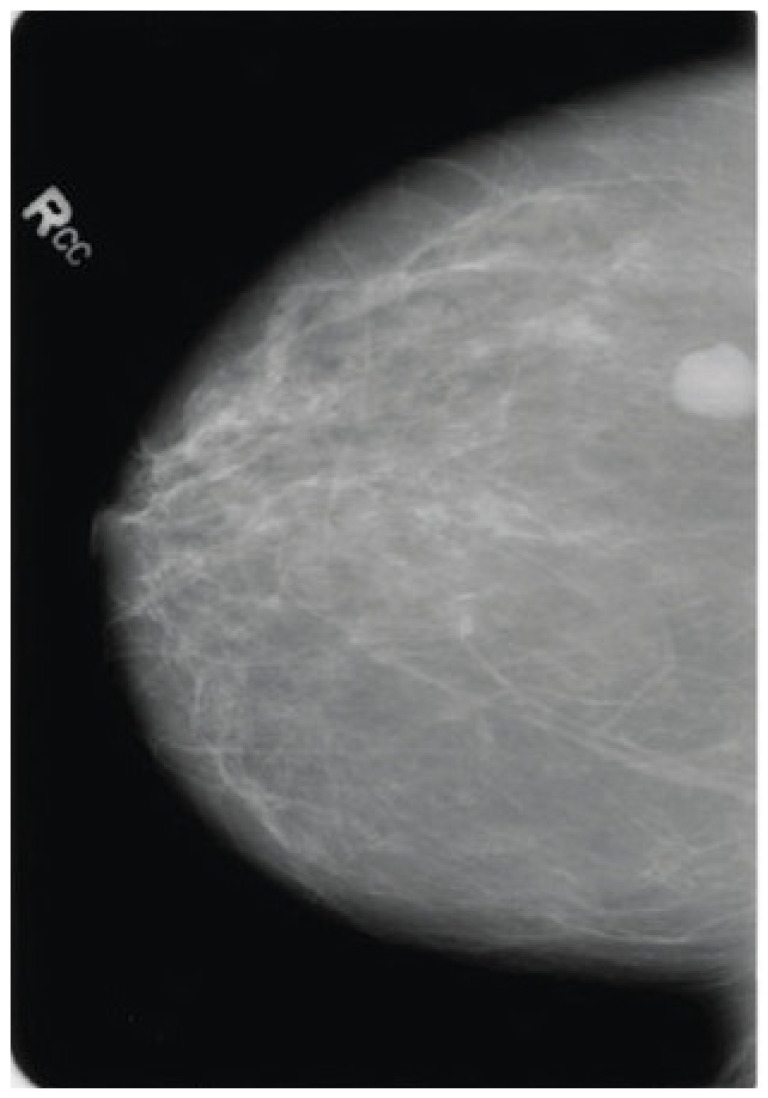	Benign	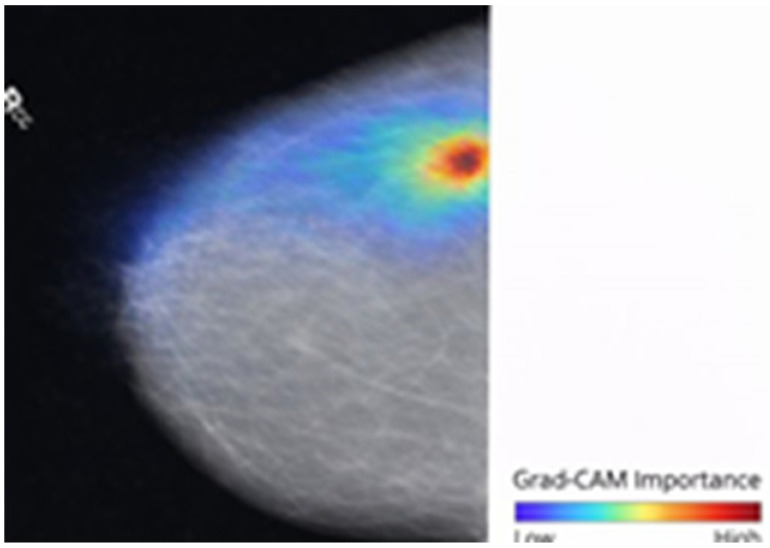	Benign	The Grad-CAM map indicates that the model correctly ignores non-malignant structures.
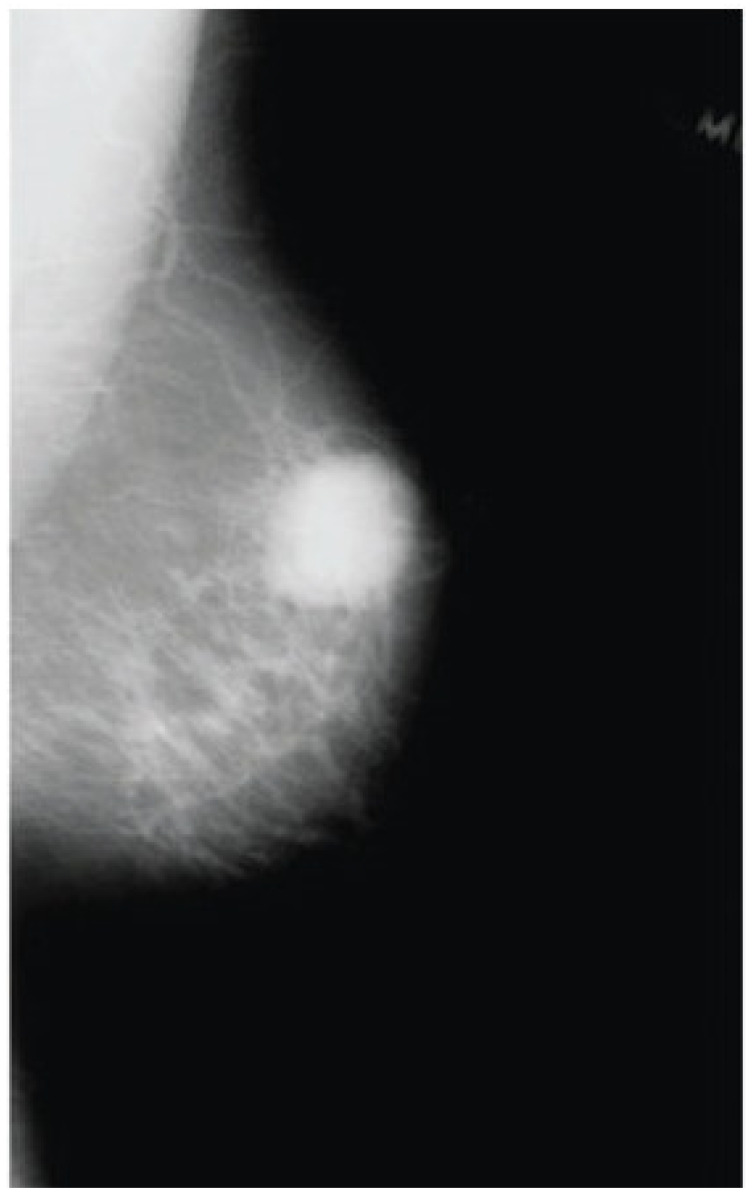	Malignant	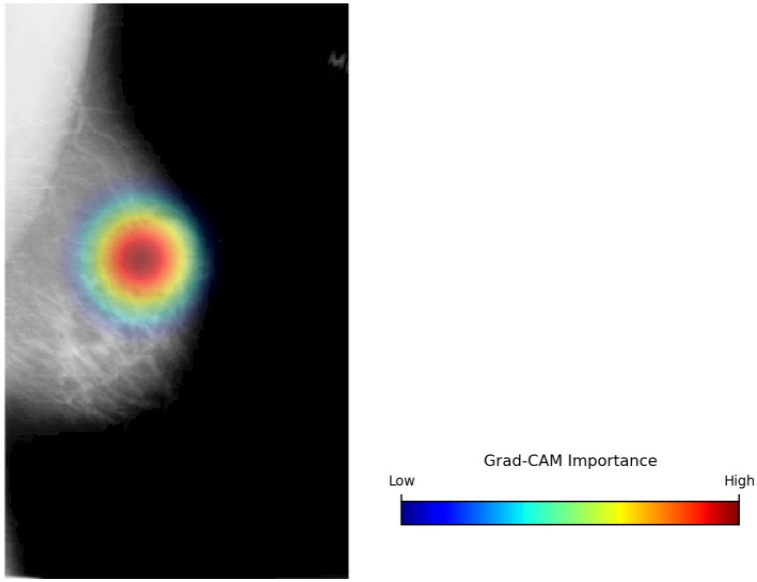	Malignant	The Grad-Cam reveals a localized activation directly over the malignant mass, identifying malignancy-related textural and structural cues.

## Data Availability

The datasets are available in the repositories: Dataset 1. Available online: https://www.cancerimagingarchive.net/collection/cbis-ddsm/ (accessed on 14 December 2025). Dataset 2. Available online: https://www.kaggle.com/datasets/ramanathansp20/inbreast-dataset (accessed on 14 December 2025).
